# mRNA melanoma vaccine revolution spurred by the COVID-19 pandemic

**DOI:** 10.3389/fimmu.2023.1155728

**Published:** 2023-03-30

**Authors:** Ziyang Xu, David E. Fisher

**Affiliations:** ^1^Department of Medicine, Massachusetts General Hospital, Boston, MA, United States; ^2^Department of Dermatology, Massachusetts General Hospital, Boston, MA, United States

**Keywords:** mRNA vaccination, COVID – 19, melanoma, cancer vaccination, neoantigen

## Abstract

The advent of mRNA vaccines represents a significant advance in the field of vaccinology. While several vaccine approaches (mRNA, DNA, recombinant protein, and viral-vectored vaccines) had been investigated at the start of the COVID-19 pandemic, mRNA vaccines quickly gained popularity due to superior immunogenicity at a low dose, strong safety/tolerability profiles, and the possibility of rapid vaccine mass manufacturing and deployment to rural regions. In addition to inducing protective neutralizing antibody responses, mRNA vaccines can also elicit high-magnitude cytotoxic T-cell responses comparable to natural viral infections; thereby, drawing significant interest from cancer immunotherapy experts. This mini-review will highlight key developmental milestones and lessons we have learned from mRNA vaccines during the COVID-19 pandemic, with a specific emphasis on clinical trial data gathered so far for mRNA vaccines against melanoma and other forms of cancer.

## 1 Introduction

For the past decade, cancer immunotherapy has been a mainstay treatment for advanced melanoma and non-small-cell lung cancer (NSLC). Reversal of the suppressive tumor microenvironment by checkpoint blockade against PD-1, PD-L1, and CTLA4 could potentiate immunosurveillance which may significantly impact clinical outcomes for patients (1). Efforts to further adjuvant the immune system through cancer vaccines had, unfortunately, yielded largely disappointing results in Phase 3 trials due to limited ability of peptide vaccines to induce CD8+ T-cell responses, induction of T cells with a restricted repertoire insufficient to counter cancer cells with heterogeneous epitope expression profiles, and inadequate induction of additional arms of the immune systems (CD4+ T cells and B cells) for synergistic tumor killing (2, 3). mRNA vaccines represent a promising strategy to tackle these challenges. They have been under development for two decades, but brought to the limelight through the COVID-19 pandemic. Massive deployment of the vaccines to billions of people in over a hundred countries across the world has modernized our infrastructure to ramp up production of such vaccines and has allowed us to gain a deep appreciation of the immune responses induced by as well as adverse effect profiles associated with mRNA vaccination in a relatively short period of time (4). Researchers and oncologists are excited to learn that mRNA vaccines can not only elicit neutralizing antibodies, commonly regarded as a key correlate of protection against SARS-CoV-2 infection, but also induce CD8+ T cell responses that mediate early protection against the virus and help surveil and eradicate tumor reservoirs in cancer patients (5). Several mRNA vaccine candidates have been advanced into clinical studies and induced positive clinical responses in several early phase clinical trials, particularly against melanoma. This review seeks to highlight lessons we have learned about mRNA vaccines during the COVID-19 pandemic and recent clinical trial data of various mRNA vaccine candidates against melanoma.

## 2 Overview of historical development of mRNA vaccine

*In vitro* transcribed (IVT) mRNAs were first used as a vector for gene transfer, whereby Wolff et al. first reported *in vivo* expression of transgenes in mouse muscles inoculated with the mRNA vector (6). Shortly after, scientists observed mRNAs encoding influenza hemagglutinin and cancer embryonic antigen (CEA) were capable of eliciting CD8+ and antigen-specific antibody responses, respectively, and started to appreciate its potential as a vector for vaccination (7, 8). However, development of the mRNA vaccines stalled in the early phase as scientists started to realize challenges associated with this platform. First, mRNA transcripts are inherently temperature-sensitive— upon dilution, they can last 6 to 12 hours at room temperature, and such cold-chain transport of the vaccines for deployment in humans can create logistical nightmares (9). Second, mRNA vaccines could trigger significant local inflammatory responses through activation of the cellular pattern recognition receptor (PRR) which can lead to dose-limiting toxicity (DLT) and significantly reduce *in vivo* transgene expression (10). Over the years, a majority of these challenges have been addressed through advanced purification and liposome formulation techniques to improve *ex vivo* and *in vivo* stability of mRNA transcripts, incorporation of modified nucleoside bases with lower likelihood of triggering PRR, and sequence-level engineering to optimize transcript stability and translation efficiency.

### 2.1 Optimization of mRNA transcripts

**Figure 1:**
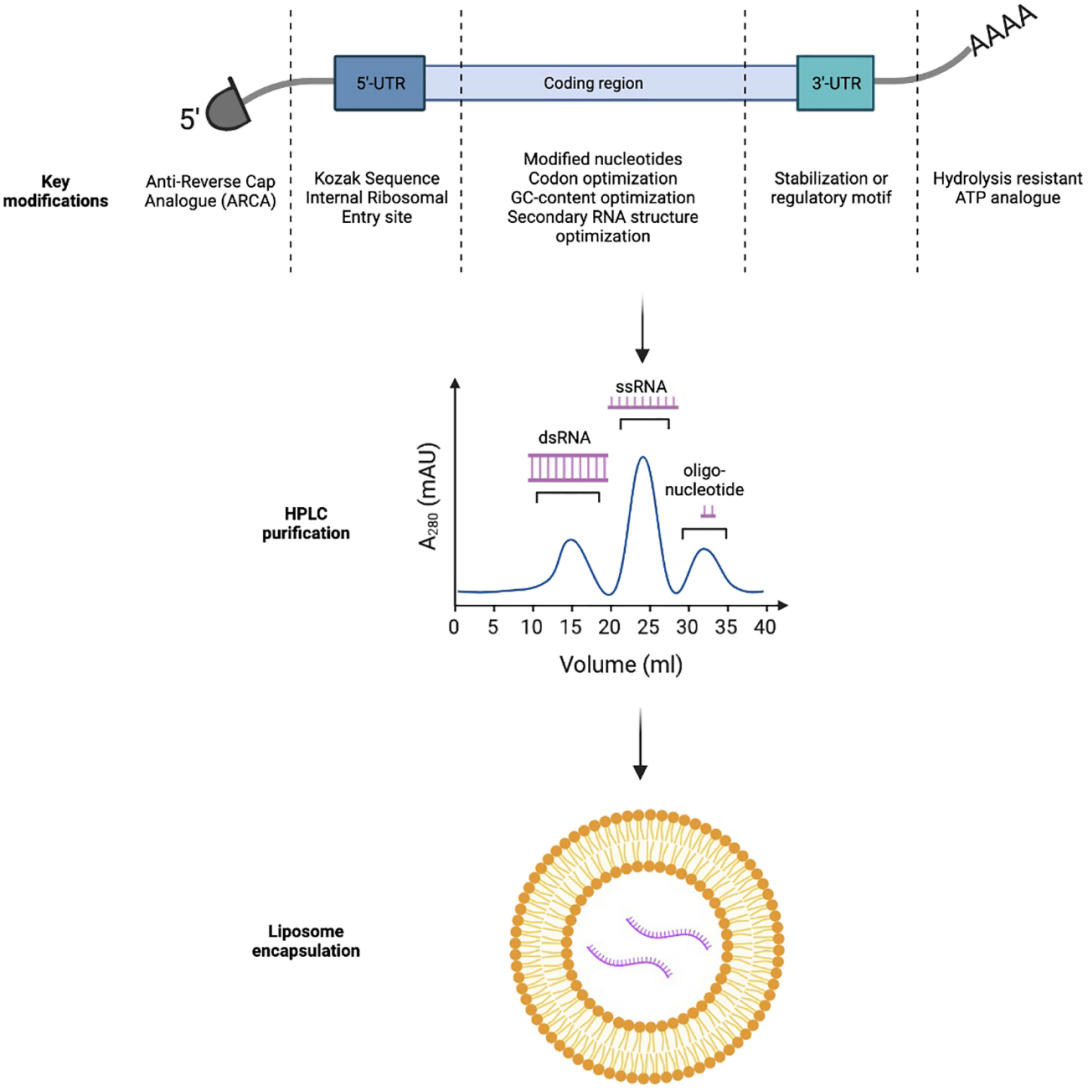
Key strategies to improve *in-vivo* expression and immunogenicity profiles of mRNA-based immunotherapeutics.

Foreign mRNAs are inherently immunogenic to the innate immune response. TLR7 and TLR8 receptors in the endosomal compartment can recognize single-stranded mRNA transcripts which are rich in unmodified guanosine and uridine-rich motifs. TLR7/8 activation can lead to type I interferon production, mediating premature degradation of the mRNA transcript and local injection reactions clinically (10). Importantly, modified nucleotides such as pseudouridine (Ψ), N6-methyladenine (m6A), 5-methylcytosine(m5C), 2-thiouracil (s2U), and 5-methyluracil (m5U) can help cloak mRNA vaccines from the innate immune system; thereby, improving the translation efficiency of the mRNA vaccines (**Figure 1**) (11, 12).

*In vitro* transcribed mRNAs frequently retain triphosphates at the 5’-end which could inadvertently trigger PRR and Type I interferon pathway activation. In eukaryotic cells, m7GpppN cap can be added to nascent mRNA transcripts through concerted actions of RNA triphosphatase, RNA guanylyltransferase, and RNA (guanine-7)-methyltransferase (13). To bypass this complex biochemical process, naturally occurring 7-methylguanosine (m7GDP) could be added directly to the *in vitro* transcription reaction mixture. Further, to avoid incorrect incorporation of m7GDP in the mRNA transcripts, anti-reverse cap analogs (ARCA) could be used alternatively to force RNA polymerase to incorporate ARCA in the forward orientation and produce fully translatable mRNA transcripts with ARCA at the 5’-end (14).

Finally, the 3’-polyA tail of *in vitro* transcribed mRNA could also be optimized. Poly-A tails are frequently added to the 3’-end of mRNA transcripts directly by RNA-polymerase or by Poly-A-polymerase. They impede RNA degradation by RNA exonucleases, significantly increasing *in vitro* and *in vivo* half-life of the transcripts. Stability of the Poly-A tail could be further improved with the use of the hydrolysis-resistant ATP analogue, ATPαS, during *in vitro* transcription. Alternatively, an oligo(dT) domain can be directly incorporated into the template DNA plasmid to precisely control the number of nucleotide bases in the Poly-A tail (15, 16).

### 2.2 Sequence-level engineering

Optimization of mRNA transcript sequence is also critical to the vector’s stability and translational efficiency. Transport RNAs (tRNAs) occur at different frequencies in different target tissues. Therefore, design of the mRNA sequence should carefully consider the mode of vaccine delivery (intradermal, intramuscular versus intravenous) to fully utilize the endogenous tRNA pool (codon usage). In addition, mRNA sequence is frequently optimized for both mouse and humans to enable preclinical evaluation of the vaccine candidates in animal models (17). In addition, increasing mRNA GC content can improve thermal stability and reduce local innate immunogenicity of the transcripts (18). Finally, secondary structures (such as stem loops and hairpins) should be minimized in the mRNA transcripts, as they can slow ribosomal scanning and reduce transgene expression (19).

The 5’ and 3’ untranslated regions (UTR) not only affect thermal stability of mRNAs but also could regulate translation of the transcripts (20). Incorporation of internal ribosomal entry sites or the Kozak sequence in the 5’-UTR can facilitate ribosomal loading and translation initiation. The 3’-UTR could either incorporate a stabilization motif such as the β-globulin 3’-UTR to prolong transcript half-life or a regulatory motif such as the miRNA-122 binding site to achieve tissue specific expression and minimize systemic toxicity by reducing off-target transgene expression in the liver (21, 22).

### 2.3 Advanced purification and formulation techniques

Double stranded RNA (dsRNA) can be either recognized by TLR3 in the endosome or RIG-I in the cytosol to trigger local type I interferon responses (23). Careful removal of the dsRNA byproducts from *in vitro* generated transcripts through High Performance Liquid Chromatography (HPLC) can thereby reduce local reactivity of mRNA vaccines (24). More recently, Baiersdorfer et al. reported the use of cellulose in ethanol-containing buffer to selectively bind dsRNA byproducts, to rapidly purify *in vitro* generated mRNA transcripts (25).

Formulation of purified mRNA transcripts has a significant influence on the transcript thermal stability (and thereby, shelf-life), transgene uptake by the target tissues, and the vaccine’s adverse effect profiles. Non-formulated (naked) mRNAs have previously been studied in several clinical trials. However, they demonstrated limited uptake and immunogenicity due to low thermal stability and poor transit across cellular membrane secondary to the negative charges on the RNA backbone (26). Self-assembled cationic polymers, such as protamine, have also been used to encapsulate negatively charged mRNAs for *in vivo* delivery. Currently, there are several protamine-formulated mRNA vaccines under clinical investigations. CV-9201, for example, is a vaccine encoding five non-small cell lung cancer (NSCLC) tumor-associated antigens (TAAs) used to treat Stage IIIb or IV NSCLC in a Phase 1/2 study. The study demonstrates that CV-9201 was well-tolerated and could induce antigen-specific T cell responses but failed to improve overall survival in vaccine recipients as compared to historical controls (27). Finally, lipid nanoparticles (LNPs) are now considered as the mainstay vector for *in vivo* mRNA delivery and have been used in both the Pfizer/Bio-N-Tech as well as the Moderna COVID-19 vaccines. LNPs utilize a mixture of cholesterol, charged lipids and polyethylene glycol (PEG) derivatives to form micelles that can stabilize negatively charged mRNA transcripts, be preferentially taken up by antigen-presenting cells (APCs) such as dendritic cells and macrophages, and offload cargos in acidic endosomes (28). In addition to being vaccine carriers, LNPs can directly serve as vaccine adjuvants through induction of IL-6 secretion, which is critical for follicular T helper (T_f_h) cell maturation (29). Furthermore, the LNPs can be further functionalized through decoration of monoclonal antibodies on their surfaces to potentiate specific targeting of these LNPs to desired cell types, thereby reducing toxicity associated with systemic administration. For example, CD5-targeted LNPs could selectively deliver mRNA to T cells for *in vivo* engineering of CAR-T cells against fibroblasts to treat heart failure in a murine model (30).

## 3 Lessons learnt from the SARS-CoV-2 pandemic

An unprecedented opportunity was created for the development of mRNA vaccines during the COVID-19 pandemic. As the original Wuhan strain was extremely contagious and associated with high mortality, academic institutions and pharmaceutical companies rapidly designed, produced and tested vaccine candidates at record speed. For the Pfizer/BioNTech vaccine, animal studies were commenced in Jan 2020 following the release of the SARS-CoV-2 genome. Phase 1/2 study initiated in April whereas Phase 2/3 study commenced in July of 2020, with the vaccine approved by the US FDA under Emergency Use Authorization (EUA) in December 2020. It was rapidly deployed to healthcare workers fighting at the frontlines against COVID-19 (31). Pre-approval clinical studies and post-marketing surveillance data generated for both Pfizer/BioNTech and Moderna vaccines were reported to be positive. For Pfizer/BioNTech vaccines, two doses of vaccine at 30 μg doses three weeks apart conferred 95% protection two months following vaccination and retained a protective efficacy of 83.7% after four months (32). The Moderna vaccine given at 100 μg dose four weeks apart conferred 94.1% protection within 64 days of vaccination and remained 90% protective after six months (33). Adverse events were mostly self-limiting, with fever and fatigue being reported as the most common events for the Pfizer vaccine in 3.8% of trial participants, and 38.1% and 15.8% of trial participants developing moderate and severe side effects after receiving the second dose of the Moderna vaccine, respectively (32, 33). Both vaccines had reduced protection against variants of concern, with the Pfizer vaccine conferring 56% and 74% protection against the omicron variant after the second and third dose respectively, due to significant mutations in the Spike protein promoting viral evasion from neutralizing antibodies generated by mRNA vaccines which were designed for the original Wuhan strain (34). Both vaccines also generated strong T-cell responses. The Moderna vaccine, for example, generated strong Th1-based CD4+ T cell responses in humans (35). While the initial analysis did not detect robust CD8+ T cell responses from the Moderna vaccine by intracellular cytokine staining (36), a subsequent study using MHC-I specific CD8 T cell sorting showed one or two doses of mRNA vaccines induced polyfunctional CD8 T cells with magnitudes comparable to natural viral infection, and with faster kinetics as compared to induction of CD4+ and neutralizing antibody responses (37). The unique ability of mRNA vaccines to induce CD8+ T-cell responses, as compared to other routes of vaccination such as protein subunit vaccines, make them an attractive platform to develop cancer vaccines.

## 4 mRNA cancer vaccines under clinical development

Currently several mRNA cancer vaccine candidates are under clinical investigation. These vaccines may target tumor associated antigens (TAA or antigens overexpressed in cancerous cells that may also be present in normal tissue), tumor-specific antigens (TSA or antigens that spontaneously arise in tumors and are therefore unique to cancer cells), or seek to prime the endogenous immune system (immunostimulants).

mRNA vaccines against TAA are currently being investigated for treatment of metastatic castration-resistant prostate cancer (NCT04382898, NCT01817738), ovarian cancer (NCT04163094), and NSCLC (NCT05142189, NCT00923312, NCT01915524) with or without CPI (NCT04382898, NCT05142189) and with or without adjuvant/neoadjuvant chemotherapies (NCT04163094, NCT05142189). NSCLC-specific mRNA vaccines CV9201 (NCT00923312) and CV9202 (NCT01915524) were found to be safe, and induced antigen-specific T-cell responses in 63% and 84% subjects respectively. However, CV9201 was not found to improve progression-free survival or overall survival in trial participants (27, 38). As TAAs are highly expressed in most cancer tissues, vaccine cocktails targeting these antigens do not need to be individualized and can be given to patients with a specific oncologic diagnosis without *a priori* knowledge of tumor transcriptomic signatures. However, as TAAs are also expressed in healthy tissues, vaccines have the theoretical risk of inducing autoimmunity (39). Central and peripheral tolerance mechanisms can also limit the magnitude of induced T cell responses (40).

To overcome these hurdles, vaccines could also be designed against TSA. For examples, vaccines could target components of oncogenic viruses (such as HPV E6 and E7 protein), which are only expressed in infected and transformed cells. BNT113 is an HPV E6/7 mRNA vaccine used for treatment of HPV-positive head and neck squamous cell carcinoma currently being studied in Phase 1 (NCT03418480) and Phase 2 (NCT04534205) trials along with PD-1 inhibitor pembrolizumab (41). TSA mRNA vaccines may also target neo-epitopes that spontaneously arise from mutational events within cancer cells. These vaccines may target cancer-driver mutations- the mRNA-5671 vaccine which targets four KRAS mutations in colorectal cancer, pancreatic cancer and NSCLC is currently being studied in a Phase I trial (NCT03948763) in combination with pembrolizumab (42). To increase the breadth of induced T-cell repertoire against tumors, these vaccines may alternatively encode a cocktail of non-driver neo-epitopes that are identified through deep sequencing of tumor exomes or transcriptomes and predicated to have high patient-specific MHC Class I binding affinity through *in silico* binding algorithms. For example, the mRNA-4650 (NCT03480152) was a personalized neo-antigen vaccine encoding up-to 20 neo-epitopes against metastatic gastrointestinal tumors. In a Phase 1 study, the vaccine induced neoantigen-specific CD8+ and CD4+ T-cell responses in three of four subjects but did not induce significant clinical responses. Further analysis showed predominant elicitation of CD4+, as opposed to CD8+, T cell responses by vaccines despite selection of HLA-I restricted epitopes during vaccine design, highlighting challenges with the *in silico* prediction algorithm (43).

mRNA can also be used as a vector for potent *in vivo* expression of biologics such as monoclonal antibodies and cytokines. mRNA encoded cytokines are often injected intratumorally to limit systemic adverse effect. For example, a Phase 1 trial investigates (NCT03739931) intra-tumoral injection of mRNA-2752 encoding three cytokines OX40L/IL23/IL36γ along with anti-PD-L1 antibody durvalumab in patients with solid tumors or lymphoma. Preliminary analyses showed dose-limiting toxicity (due to cytokine release syndrome) in one of thirty patients receiving 8mg mRNA-2752, increased cytokine IFNγ and TNFα expression in both tumor and plasma, and partial responses in two of the forty-five patients, highlighting some potential (as well as limitations) of this approach (44).

## 5 Development of mRNA vaccines against melanoma

Melanoma arises from pigment-producing melanocytes and is the most aggressive form of skin cancer. It is the 5^th^ most common form of cancer in the US and is affected by both environmental (exposure to UV radiation) and host (pigmentation characteristics, immunosuppression, hereditary) factors (45). While the early form of melanoma is readily curable through resection, the five-year survival rate for Stage 4 melanoma is only 34% (46), although recent therapeutic progress has begun to improve prognosis. As cutaneous melanoma typically harbors a relatively high mutational burden (and therefore, potentially immunogenic neo-epitopes) and is readily accessible, multiple immunotherapies have been developed in the past decade. CPI has been shown to significantly prolong survival in patients with advanced melanoma and is approved as single agent and in combination approaches by the FDA (47). Several attempts had been made to develop melanoma specific mRNA vaccines to further improve the efficacy of CPI.

Preclinical development of mRNA melanoma vaccines has been extensive. In mice, orthotopic models using B16F10 melanoma cells have been used to test vaccine candidates (48). Kreiter et al. developed an mRNA vaccine, which harbors multiple MHC class I/II-restricted neoepitopes sequenced from B16F10 cells (49). The vaccine induced potent tumor-specific CD4+ and CD8+ T-cell responses in mice and protected 60-80% from lethal tumor challenge. More recently, Chen et al. reported a novel formulation of LNP, called 113-O12B, with improved trafficking to lymph nodes as compared to liver. 113-O12 encapsulated mRNA vaccine encoding Trp2 180-188 epitope conferred complete response in 40% of mice challenged with B16F10 melanoma cells (50).

Currently, there are several LNP-formulated mRNA melanoma vaccines in clinical trial (**Table 1**). BNT111 is one of the lead candidates by BioNTech targeting melanoma tumor-associated antigens (NY-ESO-1, MAGE-C3, Tyrosinase and TPTE) currently in a Phase 2 trial (NCT04526899). In the prior Phase 1 trial, BNT111 was found to induce both CD4+ and/or CD8+ T cell responses in 39 of 50 patients (78%). In one arm where checkpoint inhibitor-experienced patients received both the vaccine and PD-1 targeting antibody cemiplimab, six of the 17 patients (35%) had partial responses to the regimen and two patients (12%) had stable disease (51). Two personalized mRNA cancer vaccines (Moderna vaccine mRNA-4157 and BioNTech vaccine BNT122) have also been advanced to Phase 2 clinical trials (NCT03815058, NCT03897881). While the data for BNT122 in melanoma is still pending at the time of this writing, the BioNTech vaccine platform attained promising results against pancreatic ductal adenocarcinoma (PDAC) in a Phase 1 trial, inducing neoantigen-specific T cell responses in 8 out of 16 participants (50%) from undetectable levels to a median of 2.9% in peripheral blood. In addition, those patients with *de-novo* immune responses had a significantly longer recurrence-free survival (RFS) (52). For mRNA-4157, the Phase 1 trial (NCT03313778) showed that the vaccine was well-tolerated and induced neoantigen-specific T cell responses. While the data has not been published in a peer-reviewed journal, Moderna and Merck recently announced that their Phase 2 trial (NCT03897881) comparing adjuvant treatment with mRNA-4157 in combination with pembrolizumab, reduced risk of recurrence or death in patients with stage 3 or 4 melanoma following complete resection, by 44% (HR=0.56 [95% CI, 0.31-1.08]) as compared to patients receiving pembrolizumab alone (54). mRNA-4157 has now been advanced to a Phase 3 trial where recruitment of participants will begin in 2023. While these various vaccines encode either TAA or TSA, non-coding RNA may also be used as adjuvant to enhance endogenous anti-tumor responses. CV8102 by CureVac consists of a non-capped, non-coding RNA complexed with a carrier peptide that is directly injected intratumorally to activate cellular TLR7/8 and RIG-1 pathways to enhance native immunity. In a Phase 1 trial (NCT03291002), CV8102 alone or in combination with CPI was observed to induce regression of injected and distant tumors in several subjects with melanoma (55).

**Table 1:** Summary of the recent clinical trial data with mRNA melanoma vaccines/immunotherapies.

Target/Administration	Clinical Trial Number	Phase	Adjuvant therapy	Adverse event profile	Efficacy Profile	Reference
BNT111 (NY-ESO-1, MAGE-C3, Tyrosinase and TPTE), given I.V.	NCT02410733	I	With or without Cemiplimab	Flu-like symptom, no dose-limiting toxicity	Expansion and activation of circulating tumor-antigen-specific T cells with memory-function and strong cytotoxic activity	(51)
	NCT04526899	II			Monotherapy: 3/25 partial response, 7/25 stable disease, 1/25 complete metabolic remission; Combined with CPI: 6/17 with partial response	
BNT122 (20 tumor-associated neo-antigens against melanoma), given I.V.	NCT03815058	I	With or without Atezolizumab	1/16 with Grade III fever/hypertension, no other Grade III or higher AE reported	*De-novo* neoantigen-specific T cell response in half (8/16) of patients, those with T-cell response had significantly longer progression-free survival than those without	(52)
BNT122 (20 tumor-associated neo-antigens against PDAC), given I.V.	NCT04161755	I				
mRNA-4157 (multiple tumor-associated neo-antigens against solid malignancy), given I.M.	NCT03313778	I	With or without pembrolizumab	No Grade III or higher AE reported; no dose-limiting toxicity observed	Neoantigen specific T cell responses have been detected by IFN-γ ELISpot from PBMCs.	(53)
mRNA-4157 (multiple tumor-associated neo-antigens against melanoma), given I.M.	NCT03897881	II	With pembrolizumab	Serious treatment-related adverse events occurred in 14.4% of patients who received the combination arm of mRNA-4157/V940 and KEYTRUDA versus 10% with KEYTRUDA alone	Adjuvant treatment of mRNA-4157 in combination with pembrolizumab reduced the risk of recurrence or death in patients with metastatic melanoma by 44% (HR=0.56 [95% CI, 0.31-1.08]) as compared to patients receiving pembrolizumab alone	(54)
CV8102 (non-coding, non-capped RNA), given intratumorally	NCT03291002	I	With or without anti-PD-1 antibodies	Most frequent AEs were Grade 1/2, including fatigue, fever, chills, and headache	Increased intra-tumoral T cell infiltration in 4/8 patients receiving CV8102 alone, and 10/18 patients receiving CV8102, and anti-PD-1 therapy	(55)
mRNA-2752 (three cytokines OX40L/IL23/IL36γ), given intratumorally in patients with lymphoma and solid tumors including melanoma	NCT03739931	I	With or without durvalumab	Dose-limiting toxicity (due to cytokine release syndrome) in one of thirty patients receiving 8 mg	Increased IFNγ and TNFα expression in both tumor and plasma; Partial responses in 2/45 patients (DLBCL and squamous-cell bladder carcinoma); 15/45 patients with stable disease	(56)

## 6 Conclusion and prospects

Since their invention three decades ago, mRNA vaccines have come a long way with multiple advances. These include modification of nucleotides, capping, sequence engineering, purification and LNP formulation, that collectively help to overcome key barriers (thermal instability and local reactogenicity) and empower the platform to be a promising tool in our fight against cancer. The COVID-19 pandemic significantly expedited RNA vaccine development, producing a deep appreciation for its immune/adverse effect profile and comfort in designing novel vaccines for quick first-in-human studies. mRNA vaccines are unique in their ability to activate multiple arms of the immune system (B cells, CD4+ and CD8+ T cells), and preliminary (not yet published) data with the Moderna melanoma neoantigen vaccine appears promising in the Phase 2 study.

However, key challenges in the field still remain. While CD8+ T cell responses are induced by the mRNA vaccines, the magnitudes of responses in humans appear to be significantly lower than those in animal studies, corresponding to more limited anti-tumor efficacy in the context of advanced disease studies to date. Further attempts to amplify cellular responses through self-amplifying RNAs (57) or antigen design through protein engineering might further improve response rates (58–60). For neoantigen-based vaccines, only a fraction of the predicted epitopes appear effective at inducing CD8+ T-cell responses (61). Ongoing improvements with *in- silico* prediction algorithms or novel *in vitro* HLA-binding assays will likely improve antigen design and best utilize the RNA cassettes. Finally, even the best-designed vaccine might not adequately overcome the suppressive tumor microenvironment at distant metastatic sites. A multimodal approach involving vaccines, CPI and immunostimulants might work synergistically (62), and should be utilized in future trial designs to attain optimal outcomes in patients with advanced melanoma or other types of malignancies, including those with lower intrinsic mutational burdens.

## Author contributions

ZX was involved in conceptualization, writing, and revision of the manuscript. DF was involved in conceptualization, writing, and revision of the manuscript. All authors contributed to the article and approved the submitted version.

## References

[1] Thallinger C Fureder T Preusser M Heller G Mullauer L Holler C. Review of cancer treatment with immune checkpoint inhibitors : Current concepts, expectations, limitations and pitfalls. Wien Klin Wochenschr (2018) 130:85–91. doi: 10.1007/s00508-017-1285-9 29098404 PMC5816095

[2] Schwartzentruber DJ Lawson DH Richards JM Conry RM Miller DM Treisman J. gp100 peptide vaccine and interleukin-2 in patients with advanced melanoma. N Engl J Med (2011) 364:2119–27. doi: 10.1056/NEJMoa1012863 PMC3517182 21631324

[3] Liu J Fu M Wang M Wan D Wei Y Wei X. Cancer vaccines as promising immuno-therapeutics: platforms and current progress. J Hematol Oncol (2022) 15:28. doi: 10.1186/s13045-022-01247-x 35303904 PMC8931585

[4] Dagan N Barda N Kepten E Miron O Perchik S Katz MA. BNT162b2 mRNA covid-19 vaccine in a nationwide mass vaccination setting. N Engl J Med (2021) 384:1412–23. doi: 10.1056/NEJMoa2101765 PMC7944975 33626250

[5] Reinscheid M Luxenburger H Karl V Graeser A Giese S Ciminski K. COVID-19 mRNA booster vaccine induces transient CD8+ T effector cell responses while conserving the memory pool for subsequent reactivation. Nat Commun (2022) 13:4631. doi: 10.1038/s41467-022-32324-x 35941157 PMC9358914

[6] Wolff JA Malone RW Williams P Chong W Acsadi G Jani A. Direct gene transfer into mouse muscle in vivo. Science (1990) 247:1465–8. doi: 10.1126/science.1690918 1690918

[7] Martinon F Krishnan S Lenzen G Magne R Gomard E Guillet JG. Induction of virus-specific cytotoxic T lymphocytes *in vivo* by liposome-entrapped mRNA. Eur J Immunol (1993) 23:1719–22. doi: 10.1002/eji.1830230749 8325342

[8] Conry RM LoBuglio AF Loechel F Moore SE Sumerel LA Barlow DL. A carcinoembryonic antigen polynucleotide vaccine for human clinical use. Cancer Gene Ther (1995) 2:33–8. 7621253

[9] Uddin MN Roni MA. Challenges of storage and stability of mRNA-based COVID-19 vaccines. Vaccines (Basel) (2021) 9 (9):1033. doi: 10.3390/vaccines9091033 34579270 PMC8473088

[10] Heil F Hemmi H Hochrein H Ampenberger F Kirschning C Akira S. Species-specific recognition of single-stranded RNA *via* toll-like receptor 7 and 8. Science (2004) 303:1526–9. doi: 10.1126/science.1093620 14976262

[11] Nance KD Meier JL. Modifications in an emergency: The role of N1-methylpseudouridine in COVID-19 vaccines. ACS Cent Sci (2021) 7:748–56. doi: 10.1021/acscentsci.1c00197 PMC8043204 34075344

[12] Morais P Adachi H Yu YT. The critical contribution of pseudouridine to mRNA COVID-19 vaccines. Front Cell Dev Biol (2021) 9:789427. doi: 10.3389/fcell.2021.789427 34805188 PMC8600071

[13] Wang SP Deng L Ho CK Shuman S. Phylogeny of mRNA capping enzymes. Proc Natl Acad Sci U.S.A. (1997) 94:9573–8. doi: 10.1073/pnas.94.18.9573 PMC23221 9275164

[14] Jemielity J Fowler T Zuberek J Stepinski J Lewdorowicz M Niedzwiecka A. Novel "anti-reverse" cap analogs with superior translational properties. RNA (2003) 9:1108–22. doi: 10.1261/rna.5430403 PMC1370475 12923259

[15] Strzelecka D Smietanski M Sikorski PJ Warminski M Kowalska J Jemielity J. Phosphodiester modifications in mRNA poly(A) tail prevent deadenylation without compromising protein expression. RNA (2020) 26:1815–37. doi: 10.1261/rna.077099.120 PMC7668260 32820035

[16] Kim SC Sekhon SS Shin WR Ahn G Cho BK Ahn JY. Modifications of mRNA vaccine structural elements for improving mRNA stability and translation efficiency. Mol Cell Toxicol (2022) 18:1–8. doi: 10.1007/s13273-021-00171-4 34567201 PMC8450916

[17] Xia X. Detailed dissection and critical evaluation of the Pfizer/BioNTech and moderna mRNA vaccines. Vaccines (Basel) (2021) 9 (7):734. doi: 10.3390/vaccines9070734 34358150 PMC8310186

[18] Park JW Lagniton PNP Liu Y Xu RH. mRNA vaccines for COVID-19: what, why and how. Int J Biol Sci (2021) 17:1446–60. doi: 10.7150/ijbs.59233 PMC8071766 33907508

[19] Wayment-Steele HK Kim DS Choe CA Nicol JJ Wellington-Oguri R Watkins AM. Theoretical basis for stabilizing messenger RNA through secondary structure design. bioRxiv (2021). doi: 10.1101/2020.08.22.262931 PMC8499941 34520542

[20] Cao J Novoa EM Zhang Z Chen WCW Liu D Choi GCG. High-throughput 5' UTR engineering for enhanced protein production in non-viral gene therapies. Nat Commun (2021) 12:4138. doi: 10.1038/s41467-021-24436-7 34230498 PMC8260622

[21] Liu T Liang Y Huang L. Development and delivery systems of mRNA vaccines. Front Bioeng Biotechnol (2021) 9:718753. doi: 10.3389/fbioe.2021.718753 34386486 PMC8354200

[22] Jain R Frederick JP Huang EY Burke KE Mauger DM Andrianova EA. MicroRNAs enable mRNA therapeutics to selectively program cancer cells to self-destruct. Nucleic Acid Ther (2018) 28:285–96. doi: 10.1089/nat.2018.0734 PMC6157376 30088967

[23] de Bouteiller O Merck E Hasan UA Hubac S Benguigui B Trinchieri G. Recognition of double-stranded RNA by human toll-like receptor 3 and downstream receptor signaling requires multimerization and an acidic pH. J Biol Chem (2005) 280:38133–45. doi: 10.1074/jbc.M507163200 16144834

[24] Kariko K Muramatsu H Ludwig J Weissman D. Generating the optimal mRNA for therapy: HPLC purification eliminates immune activation and improves translation of nucleoside-modified, protein-encoding mRNA. Nucleic Acids Res (2011) 39:e142. doi: 10.1093/nar/gkr695 21890902 PMC3241667

[25] Baiersdorfer M Boros G Muramatsu H Mahiny A Vlatkovic I Sahin U. A facile method for the removal of dsRNA contaminant from *In vitro*-transcribed mRNA. Mol Ther Nucleic Acids (2019) 15:26–35. doi: 10.1016/j.omtn.2019.02.018 30933724 PMC6444222

[26] Pardi N Hogan MJ Porter FW Weissman D. mRNA vaccines - a new era in vaccinology. Nat Rev Drug Discovery (2018) 17:261–79. doi: 10.1038/nrd.2017.243 PMC5906799 29326426

[27] Sebastian M Schroder A Scheel B Hong HS Muth A von Boehmer L. A phase I/IIa study of the mRNA-based cancer immunotherapy CV9201 in patients with stage IIIB/IV non-small cell lung cancer. Cancer Immunol Immunother (2019) 68:799–812. doi: 10.1007/s00262-019-02315-x 30770959 PMC11028316

[28] Tenchov R Bird R Curtze AE Zhou Q. Lipid nanoparticles horizontal line from liposomes to mRNA vaccine delivery, a landscape of research diversity and advancement. ACS Nano (2021) 15:16982–7015. doi: 10.1021/acsnano.1c04996 34181394

[29] Alameh MG Tombacz I Bettini E Lederer K Sittplangkoon C Wilmore JR. Lipid nanoparticles enhance the efficacy of mRNA and protein subunit vaccines by inducing robust T follicular helper cell and humoral responses. Immunity (2021) 54:2877–2892.e7. doi: 10.1016/j.immuni.2021.11.001 34852217 PMC8566475

[30] Rurik JG Tombacz I Yadegari A Mendez Fernandez PO Shewale SV Li L. CAR T cells produced *in vivo* to treat cardiac injury. Science (2022) 375:91–6. doi: 10.1126/science.abm0594 PMC9983611 34990237

[31] Mulligan MJ Lyke KE Kitchin N Absalon J Gurtman A Lockhart S. Phase I/II study of COVID-19 RNA vaccine BNT162b1 in adults. Nature (2020) 586:589–93. doi: 10.1038/s41586-020-2639-4 32785213

[32] Polack FP Thomas SJ Kitchin N Absalon J Gurtman A Lockhart S. Safety and efficacy of the BNT162b2 mRNA covid-19 vaccine. N Engl J Med (2020) 383:2603–15. doi: 10.1056/NEJMoa2034577 PMC7745181 33301246

[33] Baden LR El Sahly HM Essink B Kotloff K Frey S Novak R. Efficacy and safety of the mRNA-1273 SARS-CoV-2 vaccine. N Engl J Med (2021) 384:403–16. doi: 10.1056/NEJMoa2035389 PMC7787219 33378609

[34] Risk M Hayek SS Schiopu E Yuan L Shen C Shi X. COVID-19 vaccine effectiveness against omicron (B.1.1.529) variant infection and hospitalisation in patients taking immunosuppressive medications: a retrospective cohort study. Lancet Rheumatol (2022) 4:e775–84. doi: 10.1016/S2665-9913(22)00216-8 PMC9381025 35991760

[35] Woldemeskel BA Garliss CC Blankson JN. SARS-CoV-2 mRNA vaccines induce broad CD4+ T cell responses that recognize SARS-CoV-2 variants and HCoV-NL63. J Clin Invest (2021) 131:e149335. doi: 10.1172/JCI149335 PMC8121504 33822770

[36] Hogan MJ Pardi N. mRNA vaccines in the COVID-19 pandemic and beyond. Annu Rev Med (2022) 73:17–39. doi: 10.1146/annurev-med-042420-112725 34669432

[37] Oberhardt V Luxenburger H Kemming J Schulien I Ciminski K Giese S. Rapid and stable mobilization of CD8(+) T cells by SARS-CoV-2 mRNA vaccine. Nature (2021) 597:268–73. doi: 10.1038/s41586-021-03841-4 PMC8426185 34320609

[38] Papachristofilou A Hipp MM Klinkhardt U Fruh M Sebastian M Weiss C. Phase ib evaluation of a self-adjuvanted protamine formulated mRNA-based active cancer immunotherapy, BI1361849 (CV9202), combined with local radiation treatment in patients with stage IV non-small cell lung cancer. J Immunother Cancer (2019) 7:38. doi: 10.1186/s40425-019-0520-5 30736848 PMC6368815

[39] Sultan H Trillo-Tinoco J Rodriguez P Celis E. Effective antitumor peptide vaccines can induce severe autoimmune pathology. Oncotarget (2017) 8:70317–31. doi: 10.18632/oncotarget.19688 PMC5642557 29050282

[40] Ugel S Peranzoni E Desantis G Chioda M Walter S Weinschenk T. Immune tolerance to tumor antigens occurs in a specialized environment of the spleen. Cell Rep (2012) 2:628–39. doi: 10.1016/j.celrep.2012.08.006 22959433

[41] Klinghammer K Saba NF Castelluci E Colevas AD Rutkowski T Greil R. 155P BNT113 + pembrolizumab as first-line treatment in patients with unresectable recurrent/metastatic HNSCC: Preliminary safety data from AHEAD-MERIT. Immuno-Oncol Technol (2022) 16:100267. doi: 10.1016/j.iotech.2022.100267

[42] Nagasaka M. ES28.04 emerging mechanisms to target KRAS directly. J Thorac Oncol (2021) 16:S96–7.

[43] Cafri G Gartner JJ Zaks T Hopson K Levin N Paria BC. mRNA vaccine-induced neoantigen-specific T cell immunity in patients with gastrointestinal cancer. J Clin Invest (2020) 130:5976–88. doi: 10.1172/JCI134915 PMC7598064 33016924

[44] Hewitt SL Bai A Bailey D Ichikawa K Zielinski J Karp R. Durable anticancer immunity from intratumoral administration of IL-23, IL-36gamma, and OX40L mRNAs. Sci Transl Med (2019) 11 (477):eaat9143. doi: 10.1126/scitranslmed.aat9143 30700577

[45] Watson M Holman DM Maguire-Eisen M. Ultraviolet radiation exposure and its impact on skin cancer risk. Semin Oncol Nurs (2016) 32:241–54. doi: 10.1016/j.soncn.2016.05.005 PMC5036351 27539279

[46] Hamid O Robert C Daud A Hodi FS Hwu WJ Kefford R. Five-year survival outcomes for patients with advanced melanoma treated with pembrolizumab in KEYNOTE-001. Ann Oncol (2019) 30:582–8. doi: 10.1093/annonc/mdz011 PMC6503622 30715153

[47] Huang AC Zappasodi R. A decade of checkpoint blockade immunotherapy in melanoma: understanding the molecular basis for immune sensitivity and resistance. Nat Immunol (2022) 23:660–70. doi: 10.1038/s41590-022-01141-1 PMC9106900 35241833

[48] Hill RP Chambers AF Ling V Harris JF. Dynamic heterogeneity: rapid generation of metastatic variants in mouse B16 melanoma cells. Science (1984) 224:998–1001. doi: 10.1126/science.6719130 6719130

[49] Kreiter S Vormehr M van de Roemer N Diken M Lower M Diekmann J. Mutant MHC class II epitopes drive therapeutic immune responses to cancer. Nature (2015) 520:692–6. doi: 10.1038/nature14426 PMC4838069 25901682

[50] Chen J Ye Z Huang C Qiu M Song D Li Y. Lipid nanoparticle-mediated lymph node-targeting delivery of mRNA cancer vaccine elicits robust CD8(+) T cell response. Proc Natl Acad Sci U.S.A. (2022) 119:e2207841119. doi: 10.1073/pnas.2207841119 35969778 PMC9407666

[51] Sahin U Oehm P Derhovanessian E Jabulowsky RA Vormehr M Gold M. An RNA vaccine drives immunity in checkpoint-inhibitor-treated melanoma. Nature (2020) 585:107–12. doi: 10.1038/s41586-020-2537-9 32728218

[52] Balachandran VP Rojas LA Sethna Z Soares K Derhovanessian E Mueller F. Phase I trial of adjuvant autogene cevumeran, an individualized mRNA neoantigen vaccine, for pancreatic ductal adenocarcinoma. J Clin Oncol (2022) 40:2516–6. doi: 10.1200/JCO.2022.40.16_suppl.2516

[53] Burris HA Patel MR Cho DC Clarke JM Gutierrez M Zaks TZ. A phase I multicenter study to assess the safety, tolerability, and immunogenicity of mRNA-4157 alone in patients with resected solid tumors and in combination with pembrolizumab in patients with unresectable solid tumors. J Clin Oncol (2019) 37:2523–3. doi: 10.1200/JCO.2019.37.15_suppl.2523

[54] Merck. Moderna and Merck announce mRNA-4157/V940, an investigational personalized mRNA cancer vaccine, in combination with KEYTRUDA® (pembrolizumab), met primary efficacy endpoint in phase 2b KEYNOTE-942 trial (2022). Available at: https://www.merck.com/news/moderna-and-merck-announce-mrna-4157-v940-an-investigational-personalized-mrna-cancer-vaccine-in-combination-with-keytruda-pembrolizumab-met-primary-efficacy-endpoint-in-phase-2b-keynote-94/.

[55] Eigentler T Bauernfeind FG Becker JC Brossart P Fluck M Heinzerling L. A phase I dose-escalation and expansion study of intratumoral CV8102 as single-agent or in combination with anti-PD-1 antibodies in patients with advanced solid tumors. J Clin Oncol (2020) 38:3096–6. doi: 10.1200/JCO.2020.38.15_suppl.3096

[56] Patel MR Bauer TM Jimeno A Wang D LoRusso P Do KT. A phase I study of mRNA-2752, a lipid nanoparticle encapsulating mRNAs encoding human OX40L, IL-23, and IL-36γ, for intratumoral (iTu) injection alone and in combination with durvalumab. J Clin Oncol (2020) 38:3092–2. doi: 10.1200/JCO.2020.38.15_suppl.3092

[57] Blakney AK Ip S Geall AJ. An update on self-amplifying mRNA vaccine development. Vaccines (Basel) (2021) 9 (2):97. doi: 10.3390/vaccines9020097 33525396 PMC7911542

[58] Xu Z Wise MC Chokkalingam N Walker S Tello-Ruiz E Elliott STC. *In vivo* assembly of nanoparticles achieved through synergy of structure-based protein engineering and synthetic DNA generates enhanced adaptive immunity. Adv Sci (Weinh) (2020) 7:1902802. doi: 10.1002/advs.201902802 32328416 PMC7175333

[59] Irvine DJ Dane EL. Enhancing cancer immunotherapy with nanomedicine. Nat Rev Immunol (2020) 20:321–34. doi: 10.1038/s41577-019-0269-6 PMC7536618 32005979

[60] Xu Z Chokkalingam N Tello-Ruiz E Wise MC Bah MA Walker S. A DNA-launched nanoparticle vaccine elicits CD8(+) T-cell immunity to promote *In vivo* tumor control. Cancer Immunol Res (2020) 8:1354–64. doi: 10.1158/2326-6066.CIR-20-0061 PMC7642117 32913042

[61] Esprit A de Mey W Bahadur Shahi R Thielemans K Franceschini L Breckpot K. Neo-antigen mRNA vaccines. Vaccines (Basel) (2020) 8 (4):776. doi: 10.3390/vaccines8040776 33353155 PMC7766040

[62] Moynihan KD Opel CF Szeto GL Tzeng A Zhu EF Engreitz JM. Eradication of large established tumors in mice by combination immunotherapy that engages innate and adaptive immune responses. Nat Med (2016) 22:1402–10. doi: 10.1038/nm.4200 PMC5209798 27775706

